# 

**Published:** 1901-09-14

**Authors:** 


### NOTICE TO CORRESPONDENTS.

All MSS., Letters, Books for Review, and other matters intended for the Editor should be addressed The Editor, "The Hospital," The Hospital Building, 28 & 29 Southampton Street, Strand, W.C.

The Editor cannot undertake to return rejected MS., even when accompanied by stamped directed envelope.

**Notice to Advertisers and Subscribers.**

All Advertisements, Orders for Copies of The Hospital, and Business Communications should be addressed to **THE MANAGER (Not to the Editor)**, The Hospital, 28 & 29 Southampton Street, Strand, London, W.C.

The Cost of Subscription, post free, is as follows:—Per week, 2½d.; per quarter, 2s. 8d.; per half-year, 5s. 3d.; per annum, 10s. 6d. Foreign:—Per annum, 15s. 2d., payable, in all cases, in advance.

**NOTICE.—Vol. XXIX. of "The Hospital" (October 1900, to March, 1901), in handsome cloth binding is now on sale at the Office of this Journal, price, 7s. 6d. Cases for binding the Numbers contained in this Volume 1s. 6d. Index to Vol. XXIX., 2½d., post free.**

**The Monthly Part for September is now ready. Price 6d., by post 8d.**

### BOOKS RECEIVED.

Baillière, Tindall and Cox.

"Lessons on Massage." By Margaret D. Palmer.

Horace Hart, Oxford.
"The Ideal Nurse." By Nurse Agnes.

Gloucester Traders' Association.
"The City of Gloucester."

John Wright and Co.
"Golden Rules of Hygiene." By F. J. Waldo, M.A., M.D.Cantab., D.P.H.

J. and A. Churchill.
"A Short Manual for Monthly Nurses." By Charles J. Cullingworth M.D., F.R.C.P. Fifth edition.

### Scraps and Gleanings.

The National Society for Prevention of Cruelty to Children has received a donation of £1,000 from Mr. P. Purvis.

The scheme of providing a sanatorium for consumptives on the southern slope of Portsdown Hill (Portsmouth), which has been allowed to languish for some time has now been revived. A sum of £5,000 is required to make a start, and an effort is to be made to secure that sum.

The Metropolitan Radical Federation at a recent meeting passed a resolution that in view of the heavy cost of housing accommodation, they were of opinion that no male adult labourer engaged and living within the metropolitan area should be employed by the L.C.C. at a lower wage than 30s. a week.

The Guardians of the Isle of Wight Union Infirmary at Parkhurst are having placed in a suitable case a handsome quilt which was knitted by Queen Victoria, and given by her to the Infirmary shortly before her death. The case is to take the form of a fire screen, so that this interesting gift may be carefully preserved.

The beautiful open spaces around Tooting, Hampstead, and other localities offer a splendid opportunity to give some of the poorest of London's children a day's outing, and recently the workers of Surrey Chapel, Blackfriars, and Mr. H. Harmsworth have each had parties of children, some quite infants, who have enjoyed a variety of games and substantial meals for one day at least.

The Dorset County Hospital is a beneficiary under the will of the late Miss E. J. Maxwell to the extent of £250.

The extension of the north-east wing of Middlesex Hospital will be complete and ready for occupation by October.

The collections on Hospital Saturday at Eastbourne this year in aid of the Princess Alice Hospital amounted to £107.

The large new baths in Kentish Town, erected by the St. Pancras Borough Council, will be ready for opening at the end of September.

£33.000 per annum is paid away by the Hearts of Oak Benefit Society as sick pay to members suffering from rheumatic and gouty affections, including sciatica.

Camberwell Borough Council is about to erect municipal Turkish baths in Old Kent Road. The cost is estimated at £3,000, and the maintenance at £400 a year.

Dover Hospital Sunday was much more successful this year from a financial point of view than it was last, a result perhaps due to the very fine weather which prevailed.

The Albert Club, Fleet Street, recently gave a day's excursion to Epping Forest to 600 crippled boys and girls who were conveyed to the Forest in brakes; the excursion was under the management of the Shaftesbury Society.

The House Committee of the Coventry and Warwickshire Hospital have had under consideration the question of purchasing a Finsen lamp for use in cases of lupus. The lamp itself is estimated to cost £30. After a discussion of the matter it has been decided to defer indefinitely its further consideration.

Sir Walter Gilbey is appealing on behalf of the Royal Agricultural Benevolent Institution to the clergy and ministers of all denominations to remember that institution in the distribution of the harvest festival offertories. The Royal Agricultural Benevolent Institution provides at present for 1,200 broken-down farmers, their wives, widows, and orphans, and at the last election could only elect 75 out of 432 candidates owing to lack of funds.

The churchyard of Stanningley, a parish in Yorkshire, requiring attention, and there being no funds to pay for the necessary labour, the vicar of the parish appealed for voluntary workers to help him to carry out the necessary improvements. Some assistance was forthcoming at first in response to the appeal, but the charm of the novelty is said to have now worn off, and the vicar is left to complete his self-imposed task unaided.

### The Student of Medicine.

### APPOINTMENTS.

**W. J. Cant**, L.R.C.P.Lond., M.R.C.S.Eng., appointed Consulting Surgeon to Lincoln County Hospital. **Francis J. H. Coutts**, M.D.Vict., appointed Medical Officer of Health for Blackpool. **W. N. Davies**, M.D., M.Ch., R.U.I., appointed Certifying Factory Surgeon for the Llantrissant and Llantwit Fardre Rural District. **A. M. Dodd**, M.R.C.S., L.R.C.P.Lond., appointed Assistant Medical Officer to the Brownlow Hill Workhouse, Liverpool. **L. Eminson**, L.F.P.S., and L.M.Glasg., appointed District Medical Officer of the Cosford Union. **D. E. Evans**, M.R.C.S., L.R.C.P.Lond., appointed Certifying Factory Surgeon for the Pontypridd Urban District. **A. C. Farquharson**, M.D.Glas., D.P.H.Camb., appointed Medical Officer of Health of the Auckland Rural District Council. **A. D. Fleming**, M.B., C.M.Edin., appointed Certifying Factory Surgeon for the Kelso District, county Roxburgh. **J. Guest Gornall**, M.A., M.B., D.P.H.Cantab., appointed Medical Officer of Health County Borough of Warrington. **John Hay**, M.D., M.R.C.S., L.R.C.P., appointed Assistant Physician to the Stanley Hospital, Liverpool, and Stipendiary Medical Officer to the Hospital for Consumption and Diseases of the Chest, Liverpool. **E. H. Hicks**, B.A.Camb., M.R.C.S.Eng., appointed Medical Officer for the Wymeswold District of the Loughborough Union. **J. R. Jeffrey**, M.B., C.M.Glas., appointed Certifying Factory Surgeon for the West Linton District of the county of Peebles. **C. F. Laing**, M.B., C.M.Glasg., appointed Medical Superintendent to Somerset and Bath Asylum. **P. Leighton**, M.B., Ch.B.Edin., appointed Certifying Factory Surgeon for the Chirnside District of the County of Berwick. **Thomas Hillhouse Livingstone**, M.B., Ch.B.Edin., appointed Medical Officer of Health for the Stanhope Rural District of the Weardale Union. **Allan Mahood**, F.R.C.S.Eng., M.B., M.Ch., appointed Medical Officer of Health to the Northam Urban District Council, Devonshire. **H. P. Miles**, M.R.C.S., L.R.C.P.Lond., appointed District Medical Officer of the Kingsbridge Union. **R. W. E. Roe**, L.R.C.P., L.R.C.S.Irel., appointed Medical Officer for the Second District of the Ongar Union. **R. A. Rutherford**, L.R.C.P., L.R.C.S.Edin., appointed Certifying Factory Surgeon for the Kinlough Dispensary District of the Ballyshannon Union. **Roland A. Stevenson**, L.R.C.P., M.R.C.S.Eng., appointed Junior Resident Medical Officer at the London Open-Air Sanatorium, Pinewood, Berkshire.

